# Improved neurologically intact survival with the use of an automated, load-distributing band chest compression device for cardiac arrest presenting to the emergency department

**DOI:** 10.1186/cc11456

**Published:** 2012-08-03

**Authors:** Marcus Eng Hock Ong, Stephanie Fook-Chong, Annitha Annathurai, Shiang Hu Ang, Ling Tiah, Kok Leong Yong, Zhi Xiong Koh, Susan Yap, Papia Sultana

**Affiliations:** 1 Department of Emergency Medicine, Singapore General Hospital, Outram Road, 169608 Singapore; 2 Department of Clinical Research, Singapore General Hospital, Outram Road, 169608 Singapore; 3 Accident & Emergency, Changi General Hospital, 529889 Singapore

## Abstract

**Introduction:**

It has been unclear if mechanical cardiopulmonary resuscitation (CPR) is a viable alternative to manual CPR. We aimed to compare resuscitation outcomes before and after switching from manual CPR to load-distributing band (LDB) CPR in a multi-center emergency department (ED) trial.

**Methods:**

We conducted a phased, prospective cohort evaluation with intention-to-treat analysis of adults with non-traumatic cardiac arrest. At these two urban EDs, systems were changed from manual CPR to LDB-CPR. Primary outcome was survival to hospital discharge, with secondary outcome measures of return of spontaneous circulation, survival to hospital admission and neurological outcome at discharge.

**Results:**

A total of 1,011 patients were included in the study, with 459 in the manual CPR phase (January 01, 2004, to August 24, 2007) and 552 patients in the LDB-CPR phase (August 16, 2007, to December 31, 2009). In the LDB phase, the LDB device was applied in 454 patients (82.3%). Patients in the manual CPR and LDB-CPR phases were comparable for mean age, gender and ethnicity. The mean duration from collapse to arrival at ED (min) for manual CPR and LDB-CPR phases was 34:03 (SD16:59) and 33:18 (SD14:57) respectively. The rate of survival to hospital discharge tended to be higher in the LDB-CPR phase (LDB 3.3% vs Manual 1.3%; adjusted OR, 1.42; 95% CI, 0.47, 4.29). There were more survivors in LDB group with cerebral performance category 1 (good) (Manual 1 vs LDB 12, P = 0.01). Overall performance category 1 (good) was Manual 1 vs LDB 10, P = 0.06.

**Conclusions:**

A resuscitation strategy using LDB-CPR in an ED environment was associated with improved neurologically intact survival on discharge in adults with prolonged, non-traumatic cardiac arrest.

## Introduction

Sudden cardiac arrest is a global concern. This can be an out-of-hospital cardiac arrests (OHCA), or cardiac arrest in a patient attending the Emergency Department (ED) or an in-hospital patient. The incidence of out-of-hospital cardiac arrest (OHCA) in USA has been estimated at 1.89/1,000 person-years and at 5.98/1,000 person-years in patients with any clinically recognized heart disease [1]. Published survival rates for OHCA in North America range from 3.0% to 16.3% [2].

The problem with standard cardiopulmonary resuscitation (STD-CPR) is that it provides only one third of normal blood supply to the brain and 10% to 20% of normal blood flow to the heart [3]. Thus there have been efforts to develop mechanical CPR as an alternative. It is also increasingly recognized that although defibrillation is the definitive treatment for ventricular fibrillation, its success is also dependent on adequate circulation [4-6]. Thus, effective CPR is often a prerequisite for effective defibrillation.

The AutoPulse™ (Revivant Corporation, Sunnyvale, CA, USA) is a non-invasive load-distributing band (LDB), CPR device that generates artificial circulation mechanically. LDB-CPR is based on the concept that distributing force over the entire chest improves the effectiveness of chest compressions by delivering more total energy to the torso, but doing so without harm because that energy is distributed over a large surface area. It can be deployed in seconds by trained emergency personnel and provides automated chest compressions at a consistent rate and depth. It is designed to be compact and easy to use by trained rescuers at a variety of skill levels. The device adjusts automatically to the size and shape of each patient and is constructed around a backboard that contains a motorized rotating shaft under microprocessor control. It utilizes a load-distributing band, which is connected to the rotating shaft to compress the chest. The band is tightened or relaxed around the chest rhythmically to provide a squeezing effect. The microprocessor is programmed to provide a consistent 20% reduction in the anterior-posterior dimension of the patient's chest during the compression phase.

Other theoretical advantages of the mechanical CPR include elimination of the rescuer fatigue factor, more consistent and reliable chest compression, and elimination of the need to stop CPR during rescuer changes and patient transfers. Additionally, distributing compressive force over the anterior chest may help to mitigate the chest wall trauma, abdominal injury and thoracic visceral injury that occur frequently during STD-CPR.

Results from clinical trials have been conflicting. In a single center, phased, before-after clinical trial with 783 patients, the addition of the AutoPulse device to an EMS system was found to improve survival to discharge for patients with OHCA [7]. However, a simultaneously reported clinical trial failed to find any significance difference between manual and LDB-CPR in their primary outcome of survival to 4 hours or survival to discharge [8]. Possible explanations for these unexpected results advanced by the authors included a Hawthorne effect for manual CPR, prolonged deployment time for the devices resulting in delayed defibrillation and enrollment bias [8]. Until now, there have not been any published clinical trials assessing the effect of LDB-CPR on survival from cardiac arrest presenting to the Emergency Department.

The purpose of this study was to compare resuscitation outcomes before and after switching from manual CPR to LDB-CPR in a multi-center Emergency Department (ED) trial.

## Materials and methods

We conducted a phased, prospective cohort evaluation with intention-to-treat analysis of adults with non-traumatic cardiac arrest conveyed to, or occurring in the ED. The intervention was a system change from manual CPR to LDB-CPR at two urban EDs. This study was approved by SingHealth Centralised Institutional Review Board (IRB), Singapore and obtained waiver of informed consent (reference: CIRB 2006/052/C).

This design was chosen because these EDs adopted LDB-CPR as a new standard of care treatment in August 2007. In the medico-legal environment at the time of the study, it was difficult to get approval for a randomized controlled clinical trial design for this study. Unfortunately at that time, there was no funding or agreement from the national ambulance service for adoption of mechanical CPR either. However a successful pilot adoption of mechanical CPR at the ED in the local context was thought to be an intermediate step for pre-hospital adoption.

Singapore is a city-state with a land area of 682.3 square kilometers and a population of 4.8 million. The population is multiracial, the major ethnic groups being Chinese, Malay and Indian. Singapore Emergency Medical Service (EMS) is activated by a universal, centralized, enhanced, 995 dispatching system run by the Singapore Civil Defense Force (SCDF) and utilizing computer-aided dispatch, medical dispatch protocols, global positioning satellite (GPS) automatic vehicle locating systems and road traffic monitoring systems.

Ambulances in Singapore are manned by specifically trained paramedics (roughly equivalent to North American EMT-I). They are able to provide basic life support (BLS) and defibrillation with automated external defibrillators (AED). They are currently not certified to perform endotracheal intubation but do give adrenaline intravenously and use laryngeal mask airway devices. Mechanical CPR is not used by EMS. Paramedics only pronounce death for obvious decapitation, rigor mortis or dependent lividity. All other cardiac arrest patients are conveyed to the ED and there is no protocol for termination of resuscitation in the field.

The EDs of Singapore General Hospital and Changi General Hospital were involved in the study. Singapore General Hospital is the largest tertiary hospital in the city. A 1600-bed in-patient facility, the hospital ED sees between 300 and 500 patients daily, and between 80 and 150 cardiac arrests per year.

Changi General Hospital (CGH) Accident and Emergency (A&E) Department provides 24-hour ED specialist cover for trauma and non-trauma medical and surgical emergencies. This is supported by 24-hour x-ray, laboratory service and in-house medical and surgical specialist cover. CGH is a 790-bed regional hospital and sees between 400 and 600 patients daily at the ED. During the period of the study, none of the hospitals involved were using hypothermia post cardiac arrest.

The study population comprised cardiac arrest patients attended by the staff of the ED over the study period. There were two phases of the study. In phase 1 (control phase), we obtained prospective control data from cardiac arrest patients attended at the ED. During this period the standard of care was manual CPR. In phase 2 (intervention phase), we adopted LDB-CPR as the new standard of care for cardiac arrest patients. The protocols for deployment of the AutoPulse followed the Richmond, Virginia strategy [7], which includes an initial period of CPR performed manually before defibrillation, while aiming for early deployment of the AutoPulse device [7]. This strategy is aimed at minimizing the period without any form of CPR while the device is being deployed, and also to minimize any period to defibrillation.

All doctors and nurses in the ED received at least 30 minutes of team training in a structured program, using the device and a manikin. Emphasis was placed on teamwork, minimal delay in applying the device and minimal interruptions to CPR (manual or mechanical) while incorporating airway interventions, defibrillation or intravenous treatment. The device would usually be applied by a doctor, assisted by a nurse [9]. At the end of the training, a skills assessment was conducted, to ensure familiarity with the protocol and achievement of the targets of < 20 seconds hands-off time and < 60 seconds from the start to application of the device.

All patients with cardiac arrest as confirmed by the absence of pulse, unresponsiveness and apnea who received either CPR and/or defibrillation were eligible. We excluded patients pronounced dead without attempting CPR, according to standard operating procedure and guidelines (decapitation, rigor mortis, dependent lividity, and known 'do not resuscitate' orders). We also excluded cardiac arrest obviously caused by major trauma, non-cardiac causes and in children below age 18 years.

The primary outcome for this study was survival to hospital discharge. The secondary outcomes were return of spontaneous circulation (ROSC), survival to hospital admission and neurological (functional) status on hospital discharge. Definition of outcomes and other variables collected followed the Utstein recommendations for reporting [10-13]. Survival to hospital discharge was defined as the patient surviving the primary event and to discharge from the hospital. Return of spontaneous circulation (ROSC) was defined as the presence of any palpable pulse, which is detected by manual palpation of a major artery. Survival to admission was defined as the admission to hospital without ongoing CPR or other artificial circulatory support. Neurological (functional) status on discharge was assessed using the Glasgow-Pittsburgh outcome categories to evaluate quality of life after successful resuscitation. The cerebral performance categories (CPCs) evaluate only the cerebral performance capabilities. The overall performance categories (OPCs) reflect cerebral and non-cerebral status, and evaluate overall performance.

Data were collected from ambulance records, computer-aided dispatch and review of ED and in-hospital case records. Information was also downloaded from routinely used critical care monitors, defibrillators, ventilators and event records. Public-accessible death certificate information was also reviewed. Where necessary, we conducted an interview of the patient or their proxy to determine their current neurological (functional) outcome status. This study had full IRB approval from the institutions involved.

The quality of CPR data (Table 1) was captured using the LIFEPAK® 12 defibrillator/monitor (Physio-Control, Redmond, WA, USA). The CODE-STAT™ Suite data review software (version 7.0, Physio-Control) is able to show the number of compressions per minute over the period of resuscitation. Ventilation rates were obtained from this software version using manual annotation of impedance channel data. In addition, from April 2008, our resuscitation quality assurance program included the use of continuous video recording of all resuscitation bays. This was used only for internal quality assurance and research, with the recording devices under lock and key and accessible only by the research team. Video recordings of all resuscitations were reviewed and timings (for example, hands-off timing) were synchronized with defibrillator data.

**Table 1:** Definition of variables used for reporting quality of chest compressions

Variables	Definition
CPR ratio	The percentage of uninterrupted CPR conducted during entire resuscitation
Compression ratio	The percentage of uninterrupted chest compressions conducted during entire resuscitation
Compression rate	The average rate at which chest compression were performed during an uninterrupted series of chest compressions per minute
Compression/minute	The average number of chest compressions delivered per minute
Ventilation rate	The average rate at which ventilations were performed during an uninterrupted period of CPR
Ventilation/minute	The average number of ventilations delivered per minute
No-flow time	The sum of all pauses between chest compressions longer than 1.5 s
No-flow ratio	Mean no-flow time divided by segment length

CPR, cardiopulmonary resuscitation

We used the following parameters to measure the quality of CPR: rate of chest compressions, CPR flow fraction and the no-flow ratio (NFR), usually for the first 5 minutes of resuscitation. The CPR flow fraction, and its counterpart, the no-flow ratio (NFR), reflect the ratio of compressions to pauses (no flow) in the CPR cycle [14]. Compression is usually defined as the fraction of time with subzero position of the sternum, while no flow is defined as all pauses between compressions longer than 1.5 sec [15]. The sum of such intervals is then divided by segment time length (for example, 5 minutes), from which the NFR can be derived. The CPR ratio refers to the percentage of uninterrupted CPR during the entire resuscitation. These research parameters were calculated from the manually reviewed and annotated defibrillator recordings using the software bundled with the defibrillators. We also verified calculations manually from review of video recordings.

This study included the following specific elements for quality assurance: development of standard protocols to perform all data collection and follow-up activities, use of standardized forms; uniform criteria for patient recruitment, standardized data processing; editing of incoming data, regular communications between the study investigators to resolve questions and internal monitoring of data collection. Additional steps to ensure data quality included range checks and verification built into the data entry system, and a sequence of logic checking and examination of variables.

The primary endpoint for this study was survival to discharge. Based on the results of the trial in Richmond, Virginia [7], to detect a 6.8% improvement in survival between LDB-CPR and manual CPR (9.7% vs. 2.9%), with a two-sided test size of 5% and a power of 90% would require 295 subjects in the LDB-CPR arm. We intended to collect a total of 900 subjects at an allocation ratio of 1:1 (450 allocated to AutoPulse-CPR and 450 to STD-CPR) with allowance for loss to follow-up and also lower survival rates expected in our local setting.

Frequency tables and descriptive statistics with 95% confidence intervals (CIs) were calculated for all outcome variables listed above. All statistical analyses were carried out on an intention-to-treat basis. Associations between treatment groups and all endpoints were analyzed using the chi-square test with odds ratios presented where applicable. For each endpoint, logistic regression was used to compare the two treatment groups, adjusting for covariates that on univariate analysis were significantly different between treatment groups at *P *< 0.10.

## Results

**Figure 1:**
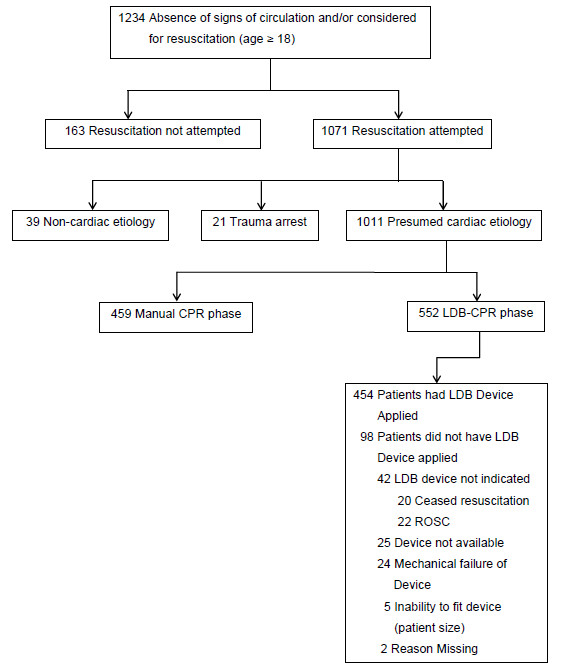
**Utstein reporting template for data elements**. LBD, load-distributing band; CPR, cardiopulmonary resuscitation; ROSC, return of spontaneous circulation.

In total, 1,011 patients were included in the study, with 459 in the manual CPR phase (1 January 2004 to 24 August 2007) and 552 patients in the LDB-CPR phase (16 August 2007 to 31 December 2009) (Figure 1).

Table 2 shows the characteristics of patients in the manual CPR and LDB-CPR phases. Patients in the two phases were comparable for mean age, gender and ethnicity. There were significant differences in arrest location, bystander CPR, initial rhythm, whether they were defibrillated and whether the arrest was bystander witnessed or EMS witnessed. These were incorporated in the logistic regression models elaborated below. More than 80% of OHCA cases had long down-times (duration from collapse to arrival at ED). The mean downtime (minutes) and standard deviation for manual CPR and LDB-CPR phases was 34:03 (16:59) and 33:18 (14:57) respectively.

**Table 2:** Characteristics of patients in the manual CPR and load distributing band CPR (LDB-CPR) phases

Characteristics	Manual CPR n = 459	LDB-CPR n = 552	*P*-value^a^
Age, years	64 (16 )	65 (16 )	0.331
Male	311 (67.8)	361 (65.4)	0.429
Race			
Chinese	310 (57.5)	369 (66.9)	
Indian	49 (10.7)	54 (9.8)	0.176
Malay	79 (17.2)	91 (16.5)	
Others	21 (4.6)	38 (6.9)	
Hospital			
Hospital A	186 (40.5)	293 (53.1)	< 0.001
Hospital B	273 (59.5)	259 (46.9)	
Medical history			
No medical history	47 (10.2)	47 (8.5)	0.347
Heart disease	155 (33.8)	192 (34.8)	0.735
Diabetes	130 (28.3)	150 (27.2)	0.685
Hypertension	198 (43.2)	251 (45.5)	0.457
Stroke	42 (9.2)	38 (6.9)	0.184
Cancer	38 (8.3)	52 (9.4)	0.526
Respiratory disease	44 (9.6)	40 (7.3)	0.180
Renal disease	22 (4.8)	58 (10.5)	0.001
Other medical history	85 (18.5)	210 (38.1)	<0.001
Unknown medical history	81 (17.7)	103 (18.7)	0.678
Arrest location			
Pre-hospital	437 (95.2)	463 (83.9)	< 0.001
Emergency Department	22 (4.8)	89 (16.1)	
Bystander witnessed	292 (63.6)	233 (42.2)	< 0.001
EMS witnessed	41 (8.9)	23 (4.2)	0.002
Bystander CPR	110 (24.0)	50 (9.1)	< 0.001
Initial rhythm			
Ventricular fibrillation	23 (5.0)	40 (7.3)	
Ventricular tachycardia	0 (0.0)	10 (1.8)	< 0.001
Asystole	340 (74.0)	336 (60.9)	
Pulseless electrical activity	80 (17.4)	119 (21.6)	
Pre-hospital defibrillation	100 (21.8)	84 (15.2)	0.007
Time of collapse to time arrived at ED (minutes)	34:03	33:18	0.591
Defibrillated at ED	124 (27.0)	154 (27.9)	0.754
AutoPulse applied	0 (0.0)	454 (82.3)	< 0.001

^a^The *t*-test was used to compare mean values and a chi-square test or Fisher's exact test was used to compare percentages. Data are presented as number (percentage) unless otherwise specified. ED, Emergency Department; CPR, cardiopulmonary resuscitation

In the LDB-CPR phase, the Autopulse was applied for 82.3% of cardiac arrest cases (Figure 1). Regarding the reasons that they were not applied for those patients, the majority were because application was not indicated on establishment of ROSC shortly after initial defibrillation with or without a brief period of manual CPR (22.4%), or because there was a valid 'do not resuscitate' order (20.4%). Other reasons for not applying were that the device was unavailable (due to being serviced or already in use) (25.5%), mechanical failure on attempted operation (24.5%) or inability to fit the device when the patient was over- or undersized (5.1%).

**Figure 2:**
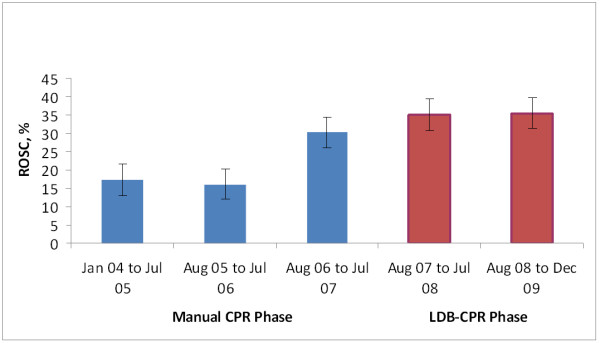
**Return of spontaneous circulation by phases**. LBD, load-distributing band; CPR, cardiopulmonary resuscitation; ROSC, return of spontaneous circulation.

**Figure 3:**
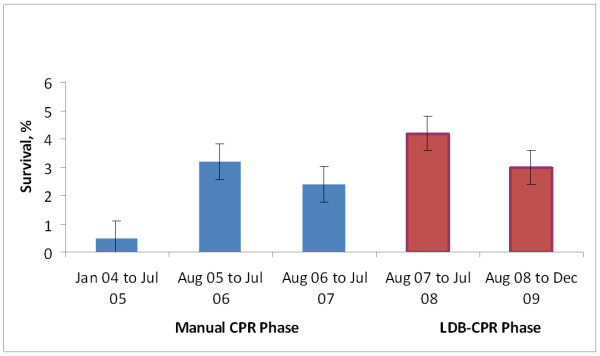
**Survival to discharge by phases**. LBD, load-distributing band; CPR, cardiopulmonary resuscitation.

Table 3 shows a comparison of outcomes in the manual CPR and LDB-CPR phases. Rates for ROSC were higher with LDB-CPR (LDB 35.3% vs. manual 22.4%, unadjusted odds ratio (OR) 1.89, 95% CI 1.43, 2.50). Using a logistic regression model for ROSC, adjusted for hospital, arrest location, bystander witnessed, EMS witnessed, initial rhythm and pre-hospital defibrillation, the OR was 1.60 (95% CI 1.16, 2.22). Figure 2 and 3 show ROSC and survival to discharge by phases.

**Table 3:** Comparison of outcomes in the manual CPR and LDB-CPR phases

	Manual CPR n = 459	LDB-CPR n = 552	Crude OR^b^ (95% CI)	Adjusted OR^c^ (95% CI)
Return of spontaneous circulation	103 (22.4)	195 (35.3)	1.89 (1.43,2.50)	1.60 (1.16, 2.22)
Survival to hospital admission	65 (14.2)	109 (19.8)	1.49 (1.07, 2.09)	1.23 (0.84, 1.81)
Survival to hospital discharge	6 (1.3)	18 (3.3)	2.55 (1.00, 6.47)	1.42 (0.47, 4.29)
Good CPC 1-2	2 (33.3%) (6 survivors)	13 (81.3%) (survivors)	8.7 (1.1, 71.6)	ND
Good OPC 1-2	2 (33.3%) (6 survivors)	12 (75.0%) (16 survivors)	6.0 (0.8, 46.1)	ND

^b^Odds ratio (OR) for the outcome for LBD-CPR versus manual CPR. ***^c^***OR was adjusted for hospital, arrest location, bystander witnessed, Emergency Medical Service (EMS) witnessed, initial rhythm, pre-hospital defibrillation, bystander cardiopulmonary resuscitation (CPR). Data are presented as number (percentage) unless otherwise specified. CPC, cerebral performance category; OPC, overall performance category. The CPC/OPC data were missing for two cases in the LDB phase. ND, not done/sample size too small.

The CPCs and OPCs for the manual CPR and LDB-CPR phases are also presented in Table 3. There were more survivors in the LDB group with CPC 1 to 2 (good) (LDB 13 vs. manual 2, OR for good CPC 8.7, 95% CI 1.1, 71.6). OPC 1 to 2 (good) was LDB 12 vs. manual 2, OR (95% CI): 6.0 (0.8, 46.1).

Table 4 shows the comparison of quality of chest compression measures in the manual and LDB phases. The mean no-flow ratio during the first 5 minutes of resuscitation was 0.29 for manual vs. 0.42 for LDB (difference = -0.13; 95% CI -0.22, -0.05). From 5 to 10 minutes into the resuscitation, the mean no-flow ratio was 0.33 for manual vs. 0.24 for LDB (difference = 0.09, 95% CI -0.01, 0.18). The mean CPR ratio for the manual group was 36 vs. 50 for LDB (difference = -14, 95% CI -24, -3).

**Table 4:** Comparison of quality of chest compression measures in the manual and load-distributing band (LDB) phases

Variables measured	Manual CPR (n = 29)	LDB CPR (n = 151)	Mean difference	**95% CI for difference**^e^
Median NFT 0 to 5 minutes (IQR), sec	84 (43-107)	114 (75-164)	-40	(-66 to -14)
Mean NFR 0 to 5 minutes (SD)	0.29 (0.17)	0.42 (0.38)	-0.13	(-0.22 to -0.05)
Median NFT 5 to 10 minutes (IQR), sec	76 (50-137)	53 (37-97)	25	(-3 to 52)
Mean NFR 5 to 10 minutes (SD)	0.33 (0.213)	0.24 (0.194)	0.09	(-0.01 to 0.18)
Mean CPR ratio (SD)	36 (21)	50 (28)	-14	(-24 to -3)
Mean compression ratio (SD)	34 (19)	46 (26)	-13	(-22 to -3)
Mean compression rate/minute (SD)	126 (14)	104 (30)	22	(11 to 33)
Mean compression/min (SD)	40 (23)	46 (28)	-6	(-17 to 5)
Mean ventilation rate/minute 0 to 5 minutes (SD)	13 (4)	8 (4)	4	(2 to 6)
Mean ventilation/minute 0 to 5 minutes (SD)	12 (4)	9 (4)	3	(2 to 5)
Mean ventilation rate/minute 5 to 10 minutes (SD)	13 (5)	7 (3)	5	(4 to 7)
Mean ventilation/minute 5 to 10 minutes (SD)	12 (5)	8 (3)	5	(3 to 6)

For elapsed time variables, median (inter-quartile range) is reported. For other variables, mean (SD) is reported.

^e^Confidence Interval; CPR, cardiopulmonary resuscitation; NFT, no-flow time; NFR, no-flow ratio.

## Discussion

In this study, we found a resuscitation strategy using LDB-CPR in an ED environment was associated with improved survival with intact neurological status on discharge in adults with non-traumatic cardiac arrest. These results provide additional evidence for the effectiveness of LDB-CPR compared to manual CPR.

In our local setting, there was unfortunately no agreement or budget for adoption of mechanical CPR by the ambulance service during the time of the study. We note that a large majority of the cases had long down-times. Paramedics in Singapore rarely pronounce death in the field, and will usually convey patients to the ED with CPR ongoing. This is reflected in the low occurrence of ventricular fibrillation or ventricular tachycardia as the ED presenting rhythm in our study (< 8%). We believe that mechanical CPR tends to be selectively used in patients with prolonged cardiac arrest rather than patients who responded immediately to initial defibrillation or CPR. These patients with prolonged cardiac arrest represent the 'excess' survivors associated with adoption of the device in the ED. Patients in prolonged cardiac arrest who are successfully resuscitated would be expected to have poorer neurological status on discharge compared to those who responded immediately, yet we found a significant improvement in neurological status with LDB-CPR in our study. This suggests that the improved cerebral perfusion obtained with LDB-CPR might actually have had a positive effect on neurological status of these survivors.

Previous animal studies could provide possible explanatory mechanisms for the observations made in this clinical observational study. In an animal study [16], LDB-CPR produced a mean coronary perfusion pressure (CPP) of 21 mm Hg compared to 14 mm Hg by manual CPR. It also produced 36% of normal coronary flow compared to 13% by manual CPR. When epinephrine was administered, LDB-CPR generated levels of flow to the heart and brain equivalent to normal flow.

In a pilot clinical study, 16 sequential terminally ill patients were enrolled from the intensive care units [17]. After 10 minutes of failed standard advanced life support (ALS) protocol, patients received alternating periods of manual CPR and LDB-CPR for 90 seconds each. LDB-CPR increased CPP more than manual chest compression (mean ± SD 20 ± 12 mmHg vs. 15 ± 11 mmHg, *P *< 0.015). LDB-CPR also increased peak aortic pressure more when compared to manual chest compression (mean ± SD 153 ± 28 mmHg versus 115 ± 42 mmHg, *P *< 0.0001).

However we have noted previously that two simultaneously reported clinical trials on the AutoPulse device in an EMS setting had differing results. One was a before-after study, which showed improved outcomes [7], while the other, a randomized controlled trial that failed to find any significance difference between manual and LDB-CPR in their primary outcome of survival to 4 hours or survival to discharge [8]. It was noted that protocols for implementation of the devices differed among the sites involved in these trials as well. In a commentary on these two studies, it was pointed out that these apparently differing results highlight the importance of the incorporation of the device into overall treatment protocols in OHCA [18], such that there is no delay in ongoing CPR or defibrillation. We believe the AutoPulse should not be seen as a replacement for manual CPR, but rather a supplemental treatment in an overall strategy for treating OHCA.

The limitations of the study need to be mentioned. This was not a randomized controlled trial but rather a before-after controlled trial, with all the limitations inherent with such a study design. For example, there was an increase in the rate of study enrollees from 10.43 per month in the control period to 19.37 during the automated CPR period. We are unable to determine if this was due to increased OHCA burden over time or increased detection and enrollment. We note that there were significant differences in the characteristics of the patients during the two phases. However the improvements in outcomes showed the same trend towards significance even after statistically controlling for these differences in a multivariate model. We are also unable to exclude that there was any 'Hawthorne' effect [19]. However we point out that as this was purely an ED study, not involving pre-hospital providers, it is unlikely that the improvement seen was due to any improvement in the quality of pre-hospital CPR.

In the initial months of the implementation of LDB-CPR, there were also some mechanical issues with the device that reduced its application. This included unfamiliarity of ED staff with the device, battery failure, device failure and limited battery life of the device. We also reported a problem of pulmonary hemorrhage when the LDB device is used together with the Oxylog (Dräger Medical AG & Co. KG) mechanical ventilator [9]. Subsequently, we changed our protocol to only use manual ventilations, prompted by the device, timed with chest compressions. There has not been any recurrence of pulmonary hemorrhage since these changes were made. Most of these mechanical issues had been resolved with the manufacturer by the end of the study.

We also noted previously that in the LDB-CPR phase, the AutoPulse was actually applied only for 82.3% of cases. A minority of instances was due to mechanical failure or device unavailability as mentioned above. However in most cases this was because application of the device was not actually indicated. For example, patients who regained a pulse soon after initial defibrillation would not have required the device. In practice, the device was usually applied in patients who did not respond to initial defibrillation and required prolonged CPR. The AutoPulse may prove to be the most useful in this setting.

Another confounding factor is that the quality of manual CPR in the control phase is based on a smaller sample of the data, as such data were not widely available during the historical control phase. In addition, due to the limited recording memory of our defibrillators, much of the quality of CPR data after office hours could not be captured. Thus what was captured may not be completely representative of the quality of CPR during the control phase. In this study, we found that there was an increase in NFR, in the first five minutes of resuscitation, with introduction of LDB-CPR into an ED. We have previously described this phenomenon [9], which emphasizes that how a device is deployed may be just as important as whether or not mechanical CPR is used. However we also found that in the subsequent five minutes of resuscitation (5 to 10 minutes), the NFT and NFR improved significantly with mechanical CPR. Overall, the NFT improved over the period of study, with training and feedback to resuscitation teams.

We acknowledge that this study is not necessarily a comparison between optimum manual CPR and mechanical CPR. However we suggest that this study would not be far removed from the usual standard of manual CPR being practiced in a real-world setting. We recommend that much attention to team training, rapid application of the device to minimize interruption and feedback from defibrillator and video recordings to improve resuscitation team performance is helpful.

We intend to follow up with this study by extending implementation of mechanical CPR to the ambulance service, to see if earlier application of such devices will make any difference in outcomes. In addition, since the trial, we have also started implementing hypothermia after cardiac arrest in the hospitals, and this may also affect neurologically intact survival.

## Conclusions

In conclusion, this is the first study to show that a resuscitation strategy using LDB-CPR in an ED environment was associated with improved neurologically intact survival on discharge in adults with prolonged, non-traumatic cardiac arrest.

This study provides further real-world evidence that adoption of this device in an ED setting can lead to improved outcomes in cardiac arrest.

## Key messages

• Rates for ROSC were higher with LDB-CPR (LDB 35.3% vs. manual 22.4%, unadjusted OR 1.89, 95% CI 1.43, 2.50).

• There were more survivors in the LDB group with CPC 1 to 2 (good) (LDB 13 vs. manual 2, OR for good CPC 8.7, 95% CI 1.1, 71.6). OPC 1 to 2 (good) was 12 for LDB vs. 2 for manual, OR 6.0, 95 CI 0.8, 46.1.

• In conclusion, this is the first study to show that a resuscitation strategy using LDB-CPR in an ED environment was associated with improved neurologically intact survival on discharge in adults with prolonged, non-traumatic cardiac arrest.

## Abbreviations

A&E: Accident and Emergency; AED: automated external defibrillators; ALS: advanced life support; BLS: basic life support; CGH: Changi General Hospital; CPC: cerebral performance category; ED: Emergency Department; IRB: institutional review board; LDB: load-distributing band; NFR: no-flow ratio; OHCA: out-of-hospital cardiac arrest; OPC: overall performance category; ROSC: return of spontaneous circulation; STD-CPR: standard cardiopulmonary resuscitation.

## Competing interests

This study was sponsored by the ZOLL Medical Corporation. The study sponsor had no involvement in the study design, data collection, data analysis and interpretation, or writing of the manuscript. Dr Ong had a patent filing related to the technology described in the study (Method of predicting acute cardiopulmonary events and survivability of a patient, Application Number: 13/047,348). Dr Ong also had a licensing agreement with the ZOLL Medical Corporation for the technology. All the other authors have neither commercial nor personal associations or any sources of support that might pose a conflict of interest in the subject matter or materials discussed in this manuscript.

## Authors' contributions

MEHO planned and established the project including the procedures for data collection, drafted the manuscript, and performed data analysis. SF and PS performed detailed statistical analysis of the data. AA drafted the manuscript, performed data collection and data analysis. SHA, LT, KLY, ZXK and SY performed data collection. All authors took part in rewriting and approved the final manuscript.
